# Application of Acupuncture to Attenuate Immune Responses and Oxidative Stress in Postoperative Cognitive Dysfunction: What Do We Know So Far?

**DOI:** 10.1155/2020/9641904

**Published:** 2020-02-13

**Authors:** Yuen-Shan Ho, Fei-Yi Zhao, Wing-Fai Yeung, Gordon Tin-Chun Wong, Hong-Qi Zhang, Raymond Chuen-Chung Chang

**Affiliations:** ^1^School of Nursing, Faculty of Health and Social Sciences, The Hong Kong Polytechnic University, Hung Hom, Kowloon, Hong Kong, China; ^2^Department of Nursing, School of International Medical Technology, Shanghai Sanda University, Shanghai, China; ^3^Department of Anaesthesiology, LKS Faculty of Medicine, The University of Hong Kong, Pokfulam, Hong Kong SAR, China; ^4^School of Chinese Medicine, Hong Kong Baptist University, Hong Kong, China; ^5^Laboratory of Neurodegenerative Diseases, School of Biomedical Sciences, LKS Faculty of Medicine, The University of Hong Kong, Hong Kong, China; ^6^State Key Laboratory of Brain and Cognitive Sciences, The University of Hong Kong, Pokfulam, Hong Kong, China

## Abstract

Postoperative cognitive dysfunction (POCD) is a common sequela following surgery and hospitalization. The prevention and management of POCD are important during clinical practice. POCD more commonly affects elderly patients who have undergone major surgery and can result in major decline in quality of life for both patients and their families. Acupuncture has been suggested as an effective intervention for many neurological disorders. In recent years, there are increasing interest in the use of acupuncture to prevent and treat POCD. In this review, we summarized the clinical and preclinical evidence of acupuncture on POCD using a narrative approach and discussed the potential mechanisms involved. The experimental details and findings of studies were summarized in tables and analyzed. Most of the clinical studies suggested that acupuncture before surgery could reduce the incidence of POCD and reduce the levels of systematic inflammatory markers. However, their reliability is limited by methodological flaws. Animal studies showed that acupuncture reduced cognitive impairment and the associated pathology after various types of surgery. It is possible that acupuncture modulates inflammation, oxidative stress, synaptic changes, and other cellular events to mitigate POCD. In conclusion, acupuncture is a potential intervention for POCD. More clinical studies with good research design are required to confirm its effectiveness. At the same time, findings from animal studies will help reveal the protective mechanisms, in which systematic inflammation is likely to play a major role.

## 1. Introduction

Postoperative cognitive dysfunction (POCD) is a common complication following surgery and hospitalization [1]. In 1955, Dr. Bedford first reported symptoms of cognitive changes and behavioral abnormalities in elderly patients who underwent general anesthesia, resulting in increasing research regarding cognitive impairment that occurs following various types of surgery, particularly cardiac surgery [2]. POCD can have profound impact on patients and their families. The most immediate effect can be observed for in-hospital education, where POCD can reduce the ability of the patient to understand and recall detailed instructions, such as wound care and drug treatment, putting the patient at higher risks of postsurgical complications. Over the long term, patients with POCD require more assistance in their daily lives than before surgery, due to the partial loss of abilities to perform normal daily activities [3]. POCD also decreases the quality of life for patients [4], and it is associated with higher mortality after surgery [5, 6]. Recently, researchers have also begun to suspect that POCD can be a harbinger for dementia because POCD and dementia share common mechanisms and there is considerable overlap in the risk factors for both diseases [7]. Alzheimer's disease, in particular, may be accelerated by POCD [8]. Therefore, prevention and management of POCD are important in clinical practice. Pharmacological interventions and advances in perioperative management are continually devloped, and their implications for POCD have been reviewed elsewhere [9, 10]. This article focuses on the use of acupuncture for POCD management. In the last decade, more than 30 clinical studies have been conducted to investigate whether acupuncture can prevent or treat POCD. A number of animal studies have also revealed the potential mechanisms underlying the effects of acupuncture during POCD prevention and treatment. In this article, we will summarize and analyze the findings from clinical trials and animal studies examining acupuncture. Thereafter, we will discuss the effects of acupuncture on inflammation, oxidative stress, synaptic changes, and other cellular events, which may potentially explain its effects during the treatment and prevention of POCD.

## 2. Effects of Acupuncture on POCD

Acupuncture has frequently been used to treat neurological and mental disorders. According to the Traditional Chinese Medicine theory, acupuncture balances the body and restores its physiological functions by stimulating specific acupoints through the insertion and manipulation of thin needles. The two most commonly used types of acupuncture techniques are manual acupuncture (MA) and electroacupuncture (EA). During MA, clinical efficacy can be achieved by lifting, thrusting, or rotating the needles by hand until “De-qi” (an irradiating feeling considered to be indicative of effective needling) is attained. During EA, electrical currents are passed through the needles, resulting in the combined therapeutic effect of MA and continuous electric pulses. Data from clinical trials and basic sciences studies suggest that MA and EA are different in terms of clinical outcomes and underlying physiology, but it is difficult to conclude which one is superior [11]. To stimulate acupoints, some researchers also use transcutaneous electrical nerve stimulation (TENS) in which low-voltage electric currents are applied to the skin surface by conducting gel pads. TENS does not involve the use of thin metal needles for stimulation. It is generally not regarded as acupuncture and therefore will not be discussed in this review. Since acupuncture is nonpharmacologically based, there are no concerns regarding dependence, addiction, tolerance, and neurological toxicity, nor will acupuncture increase the metabolic burdens of the liver and kidney, making acupuncture a potentially attractive therapy for treating POCD.

## 3. Evidence from Clinical Trials

Many clinical trials have been conducted to investigate whether acupuncture is beneficial for POCD. All of the studies were conducted in China, and most of them were published in Chinese within the last 10 years in line with the increased awareness and concern regarding POCD. As this is not a systematic review, a narrative summary on the findings followed by comments on the methodology of these studies will be provided.  Table 1 summarizes the main findings from these clinical studies. We have classified the studies into two categories, according to the time when acupuncture treatment was given and when POCD was diagnosed.

In the first category, researchers focused on the preconditioning effects of acupuncture and asked whether acupuncture pretreatment could reduce the incidence of POCD. The majority of the studies listed in Table 1 belong to this category. Acupuncture was given to the patients either before surgery or was started before and then continued throughout the entire operation. We also included a subgroup under this category, in which the acupuncture session started after the surgery (usually on the next day) and was continued daily for a few days. We considered this to be a preconditioning protocol because acupuncture was applied before any diagnosis of POCD. The timing of POCD development in these patients was also unclear; however, acupuncture was given to all patients in the study, regardless of a POCD diagnosis. The acupuncture treatment protocol varied greatly among the studies, and it is difficult to conclude which protocol provided the best results. However, almost all of the studies reported a positive effect for the treatment, except those conducted by Yang et al. [12] and Zhou et al. [13]. The incidence of POCD was evaluated for one week in most of the studies and was reported to be significantly lower in the acupuncture groups than the control groups. The long-term effects of acupuncture remain unclear, as only a few studies included follow-up periods longer than 2 weeks. Overall, DU20 (Baihui), PC6 (Neiguan), and ST36 (Zusanli) were the most commonly used acupoints for POCD pretreatments.

In the second category, there was only one study identified that focused on whether acupuncture can treat POCD [14]. Acupuncture was given to patients who had been diagnosed with POCD and were receiving conventional rehabilitation treatments. The authors reported that patients who received acupuncture in addition to usual rehabilitation treatments had higher Mini-Mental State Examination (MMSE) scores than those who only received the usual rehabilitation treatments. However, this study focused on patients who received surgery to treat traumatic brain injury, which is well-known to cause cognitive dysfunction. The results of this study must therefore be carefully interpreted, as it includes more confounding variables, such as the types and severity of the underlying traumatic brain injury. Since no detailed descriptions of the underlying injuries were provided in the paper, the reliability of the results remains questionable [14].

Although the data from clinical studies appears to be promising, it is too early to draw any conclusions. We found that many reports have missing components in their methodology. Many of them did not clearly describe how randomization was performed, nor did they describe their blinding methods. These issues could lead to potential bias and placebo effects. Some important variables for the patients, such as the number of years of education, social activities, and the presence of chronic illnesses, were not documented. All of these variables should be taken into consideration when the data are analyzed. Mood changes, such as surgery-associated anxiety, in particular, can also affect preoperative performance [15]. Therefore, mood and anxiety scales, together with the neuropsychological tests, should be administered prior to surgery to allow the statistical adjustment of cognitive test scores according to the mood state of subjects[16]. Many studies did not examine this important covariant.

Another common problem was the use of the MMSE as the only outcome measure for cognitive performance. The diagnosis of POCD should be verified by psychometric tests, comparing pre- and postoperative cognitive performances. Although there is no gold standard test for the assessment of POCD, a number of neuropsychological tests have been recommended [4], including the Montreal Cognitive Assessment (MoCA) for global cognitive change, the Digit Span Forward and Backward test, the Stroop Color Word test, and the Trail Making A & B test for executive function [4, 17, 18]. Although the MMSE test is sometimes used to quantify POCD, it is not recommended for POCD as it has a marked learning effect [19]. In fact, the MMSE test has two versions, and the use of these parallel versions in conjunction can reduce potential learning effects, resulting in greater sensitivity for the detection of functional changes associated with surgery. However, none of the studies included in Table 1 described the use of the parallel version. Compared with the MoCA, the sensitivity of the MMSE for mild cognitive changes is lower. Therefore, a more comprehensive neuropsychological battery should be used in the future for this type of study. An important issue for the detection of POCD is the standardization cognitive test administration across occasions and subjects. These tests should be administered to all subjects by the same suitably qualified and trained staff to minimize subjectivity and improve reliability [19]. Unfortunately, this standardization was likely not addressed in most of the studies described here.

The timing of postoperative cognitive tests is also a crucial element. After surgery, postoperative pain, opioids, sleep disturbance, nausea, limited mobility, and fatigue are common during the immediate postoperative period and can affect cognitive performance. Therefore, some researchers have argued that patients should not be evaluated for POCD until at least one week after the operation. However, no consensus on this issue has been reached, as findings suggest that limiting the screening period for POCD to seven days after surgery could result in missed POCD diagnoses in many surgical patients [20]. While most of the studies only detected POCD within 1 week after surgery, there is limited data regarding intermediate POCD, which may occur within 3 months, and long-term POCD, for changes 1-2 years following surgery. More studies are necessary to provide evidence on the long-term effects of acupuncture on POCD.

## 4. Potential Mechanisms of Acupuncture against POCD

Studying the mechanisms underlying the effects of acupuncture on the development of POCD in randomized, controlled trials may be challenging. To improve our understanding, animal studies may be a good option. Together with clinical findings, animal studies can provide insights for the future direction of research. We have summarized the findings of animal studies in Table 2, and we will discuss the potential mechanisms underlying the effects of acupuncture on POCD.

### 4.1. Attenuation of Systemic Inflammation and Neuroinflammation

Inflammation appears to play a major role in the development of POCD. Both systemic inflammation and neuroinflammation, particularly in the hippocampus, triggered by peripheral surgery trauma or anesthesia, have been proposed to be involved in the observed cognitive deficits [21–24]. Elevated levels of proinflammatory cytokines, such as interleukin- (IL-) 6, IL-1*β*, and tumor necrosis factor (TNF)-*α*, have been reported after surgery and may be related to POCD [25]. For instance, Xu et al. observed elevated levels of IL-6 following abdominal surgery in the elderly, which positively correlated with decline in cognitive function [26]. Geng et al. reported increased levels of IL-6, IL-1*β*, and TNF-*α* among patients undergoing laparoscopic cholecystectomy, and these increases appeared to be correlated with the choice of anesthetic agent and the incidence of POCD [27]. Consistent findings have also been reported for other types of surgery, where the levels of TNF-*α* and IL-6 in the perioperative period have been positively correlated with the development of POCD in aged patients [28, 29]. IL-1*β* and its upstream marker, TNF-*α*, are released from phagocytes and endothelial cells following tissue trauma [30]. Elevated levels of IL-1*β* can interfere and inhibit hippocampal long-term potentiation (LTP), a primary cellular mechanism that underlies memory and learning [31, 32]. IL-1*β* can also enhance glutamate neurotoxicity, which is related to cognitive dysfunctions [32].

The effects of acupuncture on inflammation have been studied extensively for various diseases and the reader if referred to the review by Park and Namgung [33]. It is therefore reasonable to speculate that acupuncture may also protect against POCD by modulating inflammatory responses, and this idea is supported by both clinical and preclinical findings.

Clinical trials have revealed that acupuncture can attenuate surgery-induced elevation of serum proinflammatory factors, such as IL-1*β*, IL-6, and TNF-*α* (please refer to Table 1). Since these cytokines are known to be associated with cognitive decline [28, 29], the clinical data appears to support a role for acupuncture in the suppression of systemic inflammation during POCD [34]. Similar findings have been observed in animals [35–37]. Rather than simply suppressing the inflammatory response, acupuncture may play a dual role in the modulation of the immune system. Data from two studies demonstrated that acupuncture reduced the levels of IL-10 (classically viewed as anti-inflammatory) and IL-6(classically viewed as proinflammatory) during the 3- to 7-day postoperative period [38, 39]. This finding is of special interest because the two cytokines are repressed simultaneously, indicating the suppression of opposing mechanisms. The plasma levels of IL-6 and IL-10 are commonly elevated after tissue damage. Some studies support the hypothesis that IL-6 and IL-10 can have both pro- and anti-inflammatory properties [40, 41]. Overall, it is not fully understood how these two cytokines, as well as other anti- and proinflammatory mechanisms, interact with each other during the postoperative period to affect cognition.

Recent studies on macrophages may further support the idea that acupuncture can result in the bidirectional modulation of the immune system. M1 and M2, two subtypes of macrophages, process proinflammatory and anti-inflammatory functions, respectively. The activation level of macrophages can affect the types and amounts of cytokines to be released. In rats with spinal cord injuries, EA treatment altered the ratio of M1/M2 macrophages. EA treatment suppressed the M1 subtypeby downregulating the marker protein CD68 and reducing TNF-*α*, IL-1*β*, and IL-6 levels, while simultaneously enhancing the proportion of M2, upregulating the marker proteins CD206 and NT-3 and increasing expression levels of IL-10 [42]. Similar findings were revealed in a study conducted by da Silva and colleagues [43], where they demonstrated that MA resulted in analgesic and anti-inflammatory effects in a rat model of muscle pain, accompanied by an M1 to M2 shift in the macrophage population and increased IL-10 expression levels. These findings are consistent with those in four other studies, where acupuncture improved cognition in rats that received various types of surgery and suppressed systemic inflammation and neuroinflammation [34].

### 4.2. Reduction of Oxidative Stress Levels

Surgical procedures inevitably produce reactive oxygen species (ROS) [44] in the body. Perioperative BBB dysfunction promotes the entry of inflammatory factors and oxidative species into the brain enabling activation of microglia and vascular endothelial cells by cytokines via surface receptors [9]. These activated microglia in turn release ROS with proinflammatory factors, creating a toxic environment in which neuronal damages are induced [9, 45]. A number of animal studies support this hypothesis. For example, elevated levels of expression of Nox2 (a key source of ROS production in the CNS) and the inflammatory cytokine IL-1*β* were found in the hippocampi of mice undergoing exploratory laparotomy. These elevated levels contributed to the production of ROS and microglial activation, eventually leading to memory dysfunction [46]. Lipid peroxidation and oxidative DNA damage can also promote neuronal dysfunction and apoptosis. Increased 8-isoprostane : creatinine ratios, an indicator of lipid peroxidation, were found in the urine of elderly patients with POCD after orthopedic surgery when compared to the non-POCD patients [47]. Moreover, researchers showed that pretreatment with the free radical scavenger edaravone resulted in a reduction in occurrence of cognitive impairment following carotid endarterectomy and spinal surgery [48, 49] suggesting that the amelioration of cerebral oxygen metabolism may be a potential management strategy for POCD.

Acupuncture has been suggested to affect oxidation levels during different medical conditions. However, most of the evidence has come from animal studies. In aged rats, EA attenuated POCD induced by acute myocardial ischemia-reperfusion, splenectomy, or partial hepatectomy. This attenuation was accompanied by reduced levels of malondialdehyde (MDA) and increased superoxide dismutase (SOD) activity in the hippocampus [34–37, 50]. A few clinical trials have also provided supportive data. A two-armed, randomized controlled trial provided direct evidence of the effect of acupuncture on oxidative stress, reporting that scalp acupuncture given before the introduction of general anesthesia in intestinal cancer patients resulted in reduced levels of MDA and SOD activity during the operation stage [51]. Indirect evidence was provided by a randomized controlled trial targeting overweight and obese subjects. Participants who received a 6-week acupuncture intervention had significantly reduced serum prooxidant/antioxidant ratio values compared with the sham acupuncture controls [52]. Similarly, in patients with rheumatoid arthritis, laser acupuncture significantly reduced plasma MDA, serum nitrate and nitrite, serum C-reactive protein, and IL-6 levels, as well as glutathione peroxidase (GPx) activity [53].

The mechanism through which acupuncture modulates the redox pathways and reduces oxidative stress remains unclear. Acupuncture likely affects a cluster of oxidative stress-related enzymes, inducing a nonspecific response [54, 55]. By using a proteomics approach, researchers found that acupuncture can induce a cluster of proteins related to oxidative stress and reduced ROS production in a rat vascular dementia model. The same study also reported decreased neuronal apoptosis and improved LTP and cell survival in the acupuncture group, suggesting the multitargeted effect of the intervention [55]. Similarly, in a study conducted by Han and colleagues, EA was found to effectively improve cognition in mice injected with lipopolysaccharide, which is widely used to induce inflammation. This group reported that EA significantly decreased MDA and hydrogen peroxide levels and increased the catalase and glutathione levels. They also reported the suppression of proinflammatory cytokine levels in the hippocampi of the treated animals [56]. Since oxidative stress and inflammation are closely related, these results are consistent with the proposed inflammation-suppressing effects of acupuncture.

### 4.3. Improvement of Synaptic Plasticity

Reduced synaptic proteins and changes in synaptic plasticity have been reported in animals after surgery. Synaptic proteins assist during the normal release of neurotransmitters and during synaptic transmission, which is critical for cognitive functions [57]. In Alzheimer's disease model transgenic mice, the levels of postsynaptic density protein 95 (PSD-95) and synaptophysin were decreased after laparotomy [58]. Exposure to high concentrations of sevoflurane was also shown to result in markedly decreased expression levels for synaptotagmin-1 in the rat hippocampus, which hindered the release of presynaptic neurotransmitters and decreased the efficiency of synaptic transmission [59]. In aged mice, laparotomy reduced the expression levels of plasticity-related proteins, such as brain-derived neurotrophic factor (BDNF), cAMP response element-binding protein (CREB), and Arc. These changes in protein levels, as well as cognitive impairments, could be attenuated by the pharmacological blockade or the genetic suppression of the inflammatory prostaglandin E2 pathway. Therefore, neuroinflammation is thought to be an upstream event of the synaptic changes observed during POCD [60].

There is little direct evidence regarding the effects of acupuncture on synaptic function during POCD. A study conducted by Xie and colleagues reported that EA can reduce impairment in spatial memory and learning and recover hippocampal LTP in aged rats that received repeated exposures to the anesthetic agent isoflurane [61]. Reports focusing on cognition-related diseases have provided indirect evidence of the effects of acupuncture. For example, when rats were injected with amyloid beta protein into their ventricles, those receiving EA showed reduced cognitive impairment and markedly different synapse morphology than those receiving no acupuncture treatment. Increased synaptic curvatures, decreased widths of synaptic clefts, and thickened postsynaptic densities were observed in the brains of rats treated with acupuncture [62]. These changes in synapse morphology may explain, at least partly, the observed cognition improving effects conveyed by EA treatment.

### 4.4. Reduction of Neuronal Injury

Neuronal damage has been suspected in humans with POCD. The S100 calcium-binding protein *β* (S100*β*) protein is a frequently used peripheral neuronal marker of neuronal damage and blood-brain barrier disruption. The protein is concentrated in glial cells and can also be detected in other nonneural cell types. During brain injury, S100*β* can leak from or be secreted by damaged cells [63]. A meta-analysis of 13 clinical studies reported that the level of serum S100*β* is correlated with the incidence of POCD [64]. Ten of the included studies reported reduced levels of serum S100*β* in the acupuncture groups [38, 39, 65–72]. Neuron-specific enolase (NSE) is another common maker used for POCD studies. NSE is an enzyme involved in the glycolytic pathway and can be found in neurons and neuroendocrine cells. Four of the included studies reported reduced levels of serum NSE in the acupuncture groups [65, 67, 68, 73]. Both S100*β* and NSE have been proposed to act as predictors of cognitive dysfunction after surgery [74].

Acupuncture provides neuroprotection through various mechanisms. The antioxidative and anti-inflammatory effects discussed above are likely to be partially responsible for its protective effects. However, acupuncture may also modulate apoptotic pathways. In aged rats that underwent splenectomy, acupuncture reduced the hippocampal Bcl-2/Bax ratio [50]. Acupuncture also increased the levels of *β*-catenin, Wnt, and glycogen synthase kinase- (GSK-) 3*β* in the hippocampi of aged rats that underwent hepatic ischemia reperfusion procedures [75], and these proteins are involved in the neuronal survival signaling pathways [76].

### 4.5. Reducing the Use of Anesthetic Agents, Promoting Patient Recovery, and Reducing Postoperative Nausea and Vomiting (PONV)

Exposure to anesthetic agents has been proposed to induce undesirable effects. Several animal studies have reported the deleterious effects of general anesthesia in the absence of surgical insults. For instance, aged rats exposed to isoflurane were found to have increased escape latencies and impaired spatial memory during the Morris water maze test. This result may be related to the metabolism of the beta-amyloid (A*β*) peptide, a key protein involved in the development of Alzheimer's disease. Isoflurane has been reported to increase the production of A*β* and promote A*β* oligomerization and accumulation in the hippocampus, which resembles the neuropathology of Alzheimer's disease [77]. Others have reported that exposure to desflurane resulted in transient spatial reference memory impairment in aged rats [78]. However, conflicting data has also been reported, showing that an anesthetic agent alone (isoflurane, sevoflurane, and propofol) was unable to impair cognition or to reduce hippocampal neurogenesis and cell survival [79–81]. Data from clinical studies have also been inconclusive, and there have been conflicting reports of the effects of anesthesia exposure on the development of POCD in elderly patients [82, 83]. There are many confounding variables, such as age, hypotension, body temperature, and hypoxia during surgery, which can make the interpretation of findings and the identification of a causal linkage between general anesthetics and cognitive and behavioral deficiencies quite challenging.

Regardless of these limitations, several studies have suggested that acupuncture could potentially reduce the required dosages of volatile anesthetic used during surgery. A meta-analysis performed by Asmussen and colleagues reported that the complementary use of acupuncture could reduce the amounts of volatile anesthetics used during craniotomy and cardiac surgery, leading to faster extubation and postoperative recovery times. They also reported a lower incidence of postoperative nausea and vomiting (PONV) in patients receiving acupuncture [84, 85]. Many of the POCD studies included in Table 1 also reported these beneficial effects of acupuncture. Reduced dosages of anesthetic agents during surgery were reported in eight studies [38, 39, 65, 66, 86–89], while a lower incidence of PONV was reported in six studies [88–93], accompanied by a lower incidence of POCD. By definition, PONV occurs during the first 24-48 h after surgery in inpatients [94]. However, a number of the studies included in Table 1 conducted their cognitive assessments during this period, and any discomfort or pain is likely to impact cognitive test performance.

Acupuncture can lead to sensations of relaxation and it may have anxiolytic properties. In addition, acupuncture may modulate the neural circuits involved in nausea. In a group of anesthetized healthy human subjects, acupuncture was reported to decrease the blood flow to the right medial frontal gyrus and to the left putamen, which are responsible for pain processing and nausea [95].

It is worth to note that pain reduction by acupuncture may also partly explain why it helps to lower the incidence of POCD. Although pain level was not assessed in most of the papers cited in this review, acupuncture is known to reduce postoperative pain [96], which is a risk factor of POCD [97]. As indicated in the systematic view conducted by Wu and colleagues in 2016 [98], acupuncture was associated with less postoperative pain one day after surgery than control treatment, although it could not reduce the use of opioid analgesic in patients. Pain research on animals showed that acupuncture could suppress inflammation and reduce the levels of reactive oxidative species. For example, in a mouse model of chronic inflammatory pain, electroacupuncture modulated the inflammatory mediators such as glial fibrillary acidic protein (GFAP) and the receptor of advanced glycation end products (RAGE) [99]. Collectively, acupuncture seems to be able to promote postoperative recovery through an array of mechanisms.

## 5. Conclusion

Based on the studies included in this review, we believe that acupuncture has the potential to be used as a complementary therapy to reduce the incidence of POCD. However, current clinical data may not be reliable as there are methodological problems for almost all of the studies, which can lead to potential bias. More clinical studies with better study designs are necessary. Regardless of the results of cognitive assessment tests, which are more susceptible to bias, the observed reductions in inflammatory and oxidative stress markers, as well as neuronal injury markers, provide solid evidence that acupuncture may protect the brain during surgery. The multiple effects of acupuncture on surgery-related conditions and mechanisms make it an interesting direction for future research. Further investigation on the types of acupuncture (MA or EA) and treatment protocol (e.g., duration, start and end time) for POCD will be clinically meaningful.

## Figures and Tables

**Table 1:** Summary of clinical trials examining the effects of acupuncture on POCD.

Author/year	Types of surgery	Study groups/no. of participants	Acupuncture intervention	Acupoints	Outcome measures	Results (compared with the control group)
Yang et al./2009 [12]	Coronary artery bypass grafting or cardiac valve replacement surgery	(i) Control: usual care/*n* = 37 (ii) Intervention: usual care+EA/*n* = 38	EA, 5 days before surgery, 30 min/day	LU2, LU7, PC6	MMSE, digit span subtest, digit symbol subtest, trail making test, short story memory test	(i) No differences in POCD incidence rates at postoperative days 7 and 14 between the two groups

Zhou et al./2011 [13]	Off-pump coronary artery bypass grafting	(i) Control: usual care+sham EA/*n* = 18 (ii) Intervention: usual care+EA/*n* = 18	EA, 30 min before surgery to the end of surgery	PC6	MMSE	(i) No differences in POCD incidence rates at the postoperative time points of 1 week and 1 month between the two groups

Zhang et al./2012 [100]	Hip or knee replacement surgery	(i) Control: usual care/*n* = 47 (ii) Intervention: usual care+EA/*n* = 41	EA, 30 min before surgery	DU20, DU24	MMSE, serum S100*β*	(i) Lower POCD incidence rate at postoperative day 1 in the intervention group (ii) No differences in postoperative serum S100*β* levels between the groups

Gao et al./2012 [92]	Noncardiac surgery	(i) Control: usual care/*n* = 60 (ii) Intervention: usual care+EA/*n* = 60	EA, 30 min before surgery to the end of surgery	DU20, PC6, ST36, LI4	MMSE, PONV incidence	(i) Incidence rates of POCD were lower at postoperative days 2 and 4 in the intervention group (ii) Lower PONV incidence in the intervention group

Lin et al./2013a [69]	Intestinal cancer resection	(i) Control: usual care/*n* = 24 (ii) EA1: usual care+EA, continuous wave, 2 Hz/*n* = 26 (iii) EA2: usual care+EA, sparse-dense wave, 2-100 Hz/*n* = 25 (iv) EA3: usual care+EA, continuous wave, 100 Hz/*n* = 24 (v) TENS: usual care+TENS, sparse-dense wave, 2-100 Hz/*n* = 25	EA/TENS, 30 min before surgery to the end of surgery	DU20, EX-HN3, PC6	MMSE, serum S100*β*	(i) Lower POCD incidence rate at postoperative day 3 in the EA and TENS group (ii) Lower postoperative serum S100*β* levels in the EA and TENS group

Lin et al./2013b [71]	Intestinal cancer resection	(i) Control: usual care/*n* = 37 (ii) Intervention: usual care+EA/*n* = 38	EA, 20 min before surgery to the end of surgery	DU20, PC6, SP6, ST36	MMSE, serum S100*β*	(i) Lower POCD incidence rate at postoperative day 3 in the intervention group (ii) Lower postoperative serum S100*β* levels in the intervention group

Lin et al./2014 [86]	Gastrointestinal cancer resection	(i) Control: usual care/*n* = 41 (ii) Intervention: usual care+EA/*n* = 42	EA, 30 min before surgery to the end of surgery	DU20, PC6, ST36	MMSE, serum IL-1*β*, IL-6, TNF-*α*	(i) Lower POCD incidence rate at postoperative day 3 in the intervention group (ii) Lower postoperative serum IL-6, IL-1*β*, and TNF-*α* levels in the intervention group (iii) Reduced amounts of anesthetic agents were used in the intervention group

Wang et al./2014 [101]	Hip replacement surgery	(i) Control: usual care/*n* = 40 (ii) Intervention: usual care+EA/*n* = 40	EA, 1 day before and 1 day after surgery, once daily, 30 min EA during surgery	Scalp acupuncture lines MS1, MS5, 2/5 middle of MS7, MS10	Neuropsychological test (did not mention the details)	(i) Lower POCD incidence rates at the postoperative time points of 6 days, 1 week, 3 months, and 6 months in the intervention group (ii) Incidence rates of POCD at the postoperative time points of 3 days, 1 week, 3 months, and 6 months were lower in the intervention group

Zhang et al./2014 [89]	Abdominal surgery	(i) Control: usual care/*n* = 60 (ii) Intervention: usual care+EA/*n* = 60	EA, 30 min before surgery to the end of surgery	DU20, DU24, PC6	MMSE, PONV incidence	(i) Lower POCD and PONV incidence rates at the postoperative time point of 48 h in the intervention group (ii) Reduced amounts of anesthetic agents were used in the intervention group (iii) Lower PONV incidence in the intervention group

Zhang et al./2014 [102]	Knee replacement surgery	(i) Control: usual care/*n* = 45 (ii) Intervention: usual care+EA/*n* = 34	EA, 30 min before surgery	DU20, DU24	MMSE, serum IL-1*β*, IL-6, TNF-*α*, S100*β*	(i) Lower POCD incidence rate at postoperative day 1 in the intervention group (ii) No differences in postoperative serum IL-1*β*, IL-6, TNF-*α*, and S100*β* levels between the groups

Zhou et al./2014 [38]	Lumbar spinal stenosis surgery	(i) Control: usual care/*n* = 30 (ii) Intervention: usual care+EA/*n* = 30	EA, 30 min before surgery to the end of surgery	LI4, PC6, SP6, ST36	MMSE, serum IL-6, IL-10, S100*β*	(i) Lower POCD incidence rate at the postoperative time point of 72 h in the intervention group (ii) Lower postoperative serum IL-6, IL-10, and S100*β* levels in the intervention group (iii) Reduced amounts of anesthetic agents were used in the intervention group

Chen/2015 [90]	Gynecological laparoscopic surgery	(i) Control: usual care/*n* = 30 (ii) Intervention: usual care+EA/*n* = 30	EA, 30 min before and 30 min after surgery	LI4, PC6	MMSE, QoR-40, serum IL-6, *β*-EP, 5-HT, PONV incidence	(i) Higher MMSE scores and lower PONV incidence at postoperative day 2 in the intervention group (ii) Higher QoR-40 scores at postoperative days 1 and 2 in the intervention group (iii) Lower postoperative serum IL-6 and 5-HT levels, higher *β*-EP levels in the intervention group (iv) Lower PONV incidence in the intervention group

Chen/2015 [65]	Laparoscopic cholecystectomy	(i) Control: usual care/*n* = 62 (ii) Intervention: usual care+EA/*n* = 62	EA, 15-30 min before surgery to the end of surgery	GB34, LI4, PC6, ST36	MMSE, serum S100*β*, NSE	(i) Lower POCD incidence rate at postoperative day 3 in the intervention group (ii) Lower postoperative serum S100*β* and NSE levels in the intervention group (iii) Reduced amounts of anesthetic agents were used in the intervention group

Jiang et al./2015 [103]	Hip or knee replacement surgery	(i) Control: usual care/*n* = 45 (ii) Intervention: usual care+EA/*n* = 43	EA, 5 days before surgery, 30 min/day	DU14, DU20	MMSE	(i) Lower POCD incidence rate at postoperative day 1 in the intervention group

Jiang/2015 [67]	Coronary artery bypass grafting	(i) Control: usual care+sham EA/*n* = 40 (ii) Intervention: usual care+EA/*n* = 40	EA, 30 min before surgery to the end of surgery	PC6	MMSE, serum S100*β*, NSE	(i) Lower POCD incidence rate at postoperative day 3 in the intervention group (ii) Lower postoperative serum S100*β* and NSE levels in the intervention group

Qing and Jiang/2015 [14]	Surgery for brain trauma	(i) Control: usual care/*n* = 80 (ii) Intervention: usual care+MA/*n* = 80	MA (scalp acupuncture) after surgery, 30 min/day for 24 days	DU17, DU24, GB13, GB19, and other scalp acupoints	MMSE	(i) Higher MMSE scores at postoperative day 24 in the treatment group

Yang et al./2015 [104]	Gastrointestinal cancer resection	(i) Normal: patients without diabetes/*n* = 45 (ii) Control: usual care/*n* = 45 (iii) Intervention: usual care+EA/*n* = 45	EA, 20 min before surgery to the end of surgery	DU20, LI10, LI11, PC6	MMSE, serum IL-1*β*, IL-6	(i) Lower POCD incidence rate at postoperative day 3 in the intervention group (ii) Serum IL-1*β* and IL-6 levels at the postoperative time points of 1 and 24 were lower in the intervention group compared with those in the control group but higher compared with those in the normal group

Zhang et al./2015 [105]	Laparoscopic cholecystectomy	(i) Control: usual care+sham EA/*n* = 35 (ii) Intervention: usual care+EA/*n* = 35	EA after surgery, 20 min/day for 7 days	DU20, PC6	MMSE	(i) Lower POCD incidence rate at postoperative days 1 and 3 in the intervention group

Dong et al./2016 [91]	Intestinal cancer resection	(i) Control: usual care/*n* = 30 (ii) Intervention: usual care+EA/*n* = 30	EA, 30 min before surgery to the end of surgery	DU20, PC6	MMSE, PONV incidence	(i) Lower POCD incidence rates at postoperative days 1 and 3 in the intervention group (ii) Lower PONV incidence rate at postoperative day 7 in the intervention group

Xie et al./2016 [106]	Hip replacement surgery	(i) Control: usual care/*n* = 60 (ii) Intervention: usual care+EA/*n* = 60	EA, 30 min before the end of surgery+after surgery, daily for 2 days, 30 min	DU24, GB13	PQRS	(i) Lower POCD incidence rates at postoperative days 1, 2, 3, and 5 in the intervention group

Yu et al./2016 [107]	Intestinal cancer resection	(i) Control: usual care/*n* = 59 (ii) Intervention: usual care+EA/*n* = 59	EA during surgery	DU20, PC6, SP6, ST36	MMSE	(i) Lower POCD incidence rate at the postoperative time points of 6 and 12 h in the intervention group

Yuan et al./2016 [72]	Extracerebral intervention	(i) Control: usual care/*n* = 61 (ii) Intervention: usual care+EA/*n* = 61	EA, 30 min before surgery	DU20, PC6, EX-HN3	MMSE, serum IL-1*β*, IL-6, TNF-*α*, S100*β*, NSE	(i) Lower POCD incidence rate at postoperative day 1 in the intervention group (ii) Lower postoperative serum IL-1*β*, IL-6, TNF-*α*, S100*β*, and NSE levels in the intervention group

Lin et al./2016 [70]	Carotid endarterectomy	(i) Control: usual care/*n* = 25 (ii) Intervention: usual care+EA/*n* = 25	EA, 30 min before surgery to the end of surgery	DU20, PC6, ST36	MoCA, plasma TNF-*α*, S100*β*, BDNF	(i) Higher MoCA scores at postoperative days 1, 3, and 7 in the intervention group (ii) Lower postoperative serum TNF-*α* and S100*β* levels in the intervention group (iii) Plasma BDNF levels in the intervention group plasma

Li/2017 [108]	Various types	(i) Control: usual care/*n* = 40 (ii) Intervention: usual care+EA/*n* = 40	EA, 30 min before surgery to the end of surgery	DU20, PC6, ST36	Serum TNF-*α*, IL-1*β*, IL-6	(i) Lower postoperative serum IL-1*β* and TNF-*α* levels in the intervention group

Li et al./2016 [68]	Hip replacement surgery	(i) Control: usual care/*n* = 42 (ii) Intervention: usual care+EA/*n* = 42	EA during surgery	MS1, MS5	Neuropsychological test, serum S100*β*, NSE	(i) Lower POCD incidence rates at the postoperative time points of 3 days, 1 week, 3 months, and 6 months in the intervention group (ii) Lower postoperative serum NES and S100*β* levels in the intervention group

Liu et al./2017 [73]	Hip replacement surgery	(i) Control: usual care/*n* = 40 (ii) Intervention: usual care+EA/*n* = 40	EA, 3 days before and 3 days after surgery, once daily, 30 min+30 min before surgery to the end of surgery	LI4, LR3	MMSE, serum IL-1*β*, TNF-*α*, S100*β*, NSE	(i) Lower POCD incidence rate at postoperative day 4 in the intervention group (ii) Lower postoperative serum IL-1*β*, TNF-*α*, NSE, and S100*β* levels in the intervention group

Liu and Teng/2017 [109]	Tumor resection	(i) Control: usual care/*n* = 49 (ii) Intervention: usual care+EA/*n* = 49	EA, 30 min before surgery to the end of surgery	DU20, PC6, ST36	MMSE, serum IL-1*β*, IL-6, TNF-*α*	(i) Lower POCD incidence rates at postoperative days 1 and 3 in the intervention group

Tao et al./2017 [110]	Knee replacement surgery	(i) Control: usual care/*n* = 30 (ii) Intervention: usual care+EA/*n* = 30	EA during surgery	DU20, DU24, PC6	MMSE	(i) Lower POCD incidence rate at postoperative day 1 in the intervention group

Wang/2017 [111]	Nonspecific type	(i) Control: usual care/*n* = 28 (ii) Intervention: usual care+EA/*n* = 29	EA, after surgery, once daily for 7 days, 30 min	DU20, PC6	MMSE, FAQ	(i) Higher MMSE and lower FAQ scores at postoperative days 1 and 3 in the intervention group

Xiao et al./2017 [88]	Cardiac valve replacement with cardiopulmonary bypass	(i) Control: usual care/*n* = 22 (ii) Intervention: usual care+EA/*n* = 22	EA, 20 min before surgery to the end of surgery	DU20, HT7, PC4, PC6	MMSE, PONV incidence	(i) Lower POCD incidence rate at postoperative day 3 in the intervention group (ii) Reduced amounts of anesthetic agents were used in the intervention group (iii) Lower PONV incidence in the intervention group

Zhang et al./2017 [93]	Cardiac valve replacement with cardiopulmonary bypass	(i) Control: usual care+sham EA/*n* = 20 (ii) Intervention: usual care+EA/*n* = 20	(i) EA, 20 min before surgery to the end of surgery	DU20, DU24, PC4, PC6	MMSE, QOR-9, PONV incidence	(i) Higher MMSE scores at postoperative days 1 and 3 in the intervention group (ii) Improved QOR-9 scores in the intervention group (iii) Lower PONV incidence in the intervention group

Zhang et al./2017 [39]	Spine surgery	(i) Control: usual care/*n* = 45 (ii) Intervention: usual care+EA/*n* = 45	EA, 30 min before surgery to the end of surgery	DU14, DU20, ST36	MMSE, serum IL-6, IL-10, S100*β*	(i) Higher MMSE scores at postoperative day 7 in the intervention group (ii) Lower postoperative serum IL-6, IL-10, and S100*β* levels in the intervention group (iii) Reduced amounts of anesthetic agents were used in the intervention group

Zhao and Li/2017 [112]	Laparoscopic cholecystectomy	(i) Control: usual care/*n* = 28 (ii) MA: usual care+MA/*n* = 29 (iii) EA: usual care+EA/*n* = 29	MA or EA after surgery, 30 min/day for 7 days	DU20, PC6	MMSE, FAQ	(i) Higher MMSE scores at postoperative days 1 and 3 in the EA and MA groups (ii) Lower FAQ scores at postoperative day 3 in the EA and MA groups

Zheng/2017 [113]	Intestinal cancer resection	(i) Control: usual care/*n* = 56 (ii) Intervention: usual care+EA/*n* = 56	EA during surgery	DU20, PC6, SP6, ST36	MMSE	(i) Lower POCD incidence rates at the postoperative time points of 6 and 12 h in the intervention group

Dong et al./2018 [114]	Hip replacement surgery	(i) Control: usual care/*n* = 20 (ii) Drug: usual care+dexmedetomidine/*n* = 20 (iii) MA: usual care+dexmedetomidine+MA/*n* = 20	MA, 20 min before surgery	DU20, LI4, PC6, ST36	MMSE	(i) Lower POCD incidence rate at postoperative day 1 in both intervention groups (ii) Lower POCD incidence rate in the MA+drug group compared with the drug-only group (iii) Incidence rates of POCD at postoperative day 1 were the lowest in the MA group and the highest in the control group

Han/2018 [66]	Intestinal cancer resection	(i) Control: usual care/*n* = 45 (ii) Intervention: usual care+EA/*n* = 45	EA SW 20 min before surgery to the end of surgery	DU20, PC6, SP6, ST36	MMSE, serum S100*β*	(i) Higher MMSE scores at the postoperative time points of 12, 24, and 36 h in the intervention group (ii) Lower postoperative serum S100*β* levels in the intervention group (iii) Reduced amounts of anesthetic agents were used in the intervention group

Liu et al./2018 [115]	Hip replacement surgery	(i) Control: usual care/*n* = 60 (ii) Intervention: usual care+EA/*n* = 60	EA, 3 days before surgery and 3 days after surgery, once daily, 30 min+30 min before surgery to the end of surgery	LI4, LR3	MMSE, serum IL-1*β*, TNF-*α*, cortisol, epinephrine, norepinephrine, CD3^+^, CD4^+^, CD8^+^, CD4/CD8 ratio, CD16^+^, CD56^+^	(i) Lower POCD incidence rate at postoperative day 4 in the intervention group (ii) Lower postoperative serum levels of IL-1*β*, TNF-*α*, cortisol, epinephrine, and norepinephrine (iii) Higher postoperative serum levels of CD3^+^, CD4^+^, CD8^+^, CD4^+^/CD8^+^, CD16^+^, and CD56^+^ in the intervention group

Sun et al./2018 [116]	Gastrectomy for gastric carcinoma	(i) Control: usual care/*n* = 20 (ii) EA: usual care+EA/*n* = 20 (iii) Sham: usual care+sham EA/*n* = 20	EA, 20 min before surgery to the end of surgery	LI4, PC6, ST36, ST37	MMSE, serum IL-6, TNF-*α*	(i) Lower POCD incidence rate at postoperative day 3 in the EA group (ii) Lower postoperative serum IL-6 and TNF-*α* levels in the intervention group

Wang et al./2018 [117]	Subtotal gastrectomy	(i) Control: usual care/*n* = 48 (ii) Intervention: usual care+EA/*n* = 48	EA, 15-20 min before surgery	LI4, PC6, ST36, ST37	MMSE, MoCA, CD3^+^, CD4^+^, CD8^+^, CD4^+^/CD8^+^ ratio	(i) A trend of higher MMSE and MoCA scores at postoperative day 1 in the intervention group (ii) Higher CD3^+^, CD4^+^, and CD4^+^/CD8^+^ levels at certain postoperative time points

Wang/2018 [87]	Nonspecific type	(i) Control: usual care/*n* = 29 (ii) Intervention: usual care+EA/*n* = 29	EA, 30 min before surgery	DU20, PC6, ST36	MMSE, serum IL-1*β*, IL-6, TNF-*α*	(i) Higher MMSE scores at postoperative day 3 in the intervention group (ii) Lower postoperative serum IL-1*β*, IL-6, and TNF-*α* levels in the intervention group (iii) Reduced amounts of anesthetic agents were used in the intervention group

Zeng and Wang/2018 [118]	Lower abdomen surgery and lower limb surgery	(i) Control: usual care/*n* = 50 (ii) Intervention: usual care+EA/*n* = 50	EA, after surgery, daily for 30 days	BL23, DU20, GB20	MMSE, acetylcholine and cholinesterase activity in cerebrospinal fluid	(i) Higher MMSE scores at postoperative days 1, 3, and 7 in the intervention group (ii) Lower POCD incidence rates at postoperative days 3 and 7 in the intervention group (iii) Higher acetylcholine levels and lower cholinesterase activities after surgery in the intervention group

Abbreviations: MA: manual acupuncture; EA: electroacupuncture; TENS: transcutaneous electrical nerve stimulation; MMSE: Mini-Mental State Examination ((preoperative scores-postoperative scores) ≥ 2 indicates occurrence of POCD); FAQ: Functional Activities Questionnaire; MoCA: Montreal Cognitive Assessment; PONV: postoperative nausea and vomiting; QoR-40: 40-item quality of recovery score; QOR-9: quality of recovery-9; PQRS: postoperative quality recovery scale; IL-1*β*: interleukin-1*β*; IL-6: interleukin-6; IL-10: interleukin-10; TNF-*α*: tumor necrosis factor-*α*; S100*β*: S100 calcium-binding protein *β*; NSE: neuron-specific enolase; BDNF: brain-derived neurotrophic factor; 5-HT: 5-hydroxy tryptophan; *β*-EP: *β*-endorphin; BL23: Shenshu; DU14: Dazhui; DU17: Naohu; DU20: Baihui; DU21: Qianding; DU24: Shenting; EX-HN3: Yintang; GB4: Hanyan; GB6: Xuanli; GB7: Qubin; GB13: Benshen; GB19: Naokong; GB20: Fengchi; GB34: Yanglingquan; HT7: Shenmen; LI4: Hegu; LI10: Shousanli; LI11: Quchi; LR3: Taichong; LU2: Yunmen; LU7: Lieque; PC4: Ximen; PC6: Neiguan; SP6: Sanyinjiao; ST36: Zusanli; ST37: Shangjuxu.

**Table 2:** Summary of animal studies examining the effects of acupuncture on POCD.

Author/year	Type of surgery	Animals and study groups/group size	Acupuncture interventions	Acupoints	Results
Ye et al./2014 [119]	Partial hepatectomy (PH)	(i) Male SD rats, 12 months old (ii) Control: no treatment/*n* = 30 (iii) PH only/*n* = 30 (iv) PH+EA/*n* = 30 (v) PH+minocycline/*n* = 30	EA, 30 min/day after surgery, for 1, 3, or 7 days	DU20, DU14	(i) EA improved performance in MWM (ii) EA reduced serum levels of CHR, ACTH, and corticosterone

Yuan et al./2014 [34]	Acute myocardial ischemia-reperfusion (AMIR)	(i) Male, SD rats, 18–24 months old (ii) Sham/*n* = 30 (iii) AMIR/*n* = 30 (iv) AMIR+EA/*n* = 30	EA, immediately after reperfusion started, for 30 min	DU20, ST36	(i) EA group showed a trend of improvement in working and reference memory in the 8-arm maze task (ii) EA improved survival rates, reduced the excitability of the sympathetic nerve, and attenuated neural apoptosis and microglial activation (Iba-1 ↓) (iii) EA suppressed hippocampal oxidative stress (MDA and SOD ↓), reduced peripheral inflammation (IL-6 and TNF-*α* ↓)

Yin et al./2015 [50]	Splenectomy (ST)	(i) Male SD rats, 20 months old (ii) Control: no procedure/*n* = 5 (iii) Sham: anesthesia only/*n* = 15 (iv) Anesthesia+ST/*n* = 15 (v) Anesthesia+ST+EA/*n* = 15	EA, 20 min before surgery	DU20, PC6, EX-HN3	(i) EA improved performance in Y-maze (ii) EA reduced serum concentration levels of IL -1*β*, IL-6, and TNF-*α*, reduced hippocampal Bcl-2/Bax ratio

Wang et al./2016 [35]	Splenectomy (ST)	(i) Male SD rats, 18–20 months old (ii) Control: no treatment/*n* = 30 (iii) ST only/*n* = 30 (iv) ST+EA sham/*n* = 30 (v) ST+EA/*n* = 30 (vi) ST+EA+AMPK inhibitor	EA, 30 min/day, 5 days before surgery	DU20	(i) EA improved performance in MWM (ii) EA reduced hippocampal expression of NF-*κβ*, IL-1*β*, and TNF*α*

Xie et al./2016 [61]	No surgery	(i) Male and female SD rats, 20 months old (ii) Control: no treatment/*n* = 16 (iii) Isoflurane only/*n* = 16 (iv) Isoflurane+EA/*n* = 16	EA, during isoflurane anesthesia for 4 h	DU20	(i) EA improved performance in MWM (ii) EA reduced hippocampal LTP lesion

Chen et al./2017 [120]	Trigeminal neuralgia (TN) model	(i) Male SD rats, 200–260 g (ii) Control: no treatment/*n* = 10 (iii) TN only/*n* = 10 (iv) TN+EA/*n* = 10 (v) TN+pregabalin/*n* = 10	EA, 30 min each time, once every 2 days for 11 consecutive days	LI10, L111	(i) EA improved performance in MWM (ii) EA reduced demyelination in the Gasserian ganglion and medulla oblongata (iii) EA reduced vacuolar degeneration and swelling of mitochondria in hippocampal neurons (iv) EA increased fEPSP slope in electrophysiological study

Chen et al./2017 [75]	Hepatic ischemia reperfusion (HIR)	(i) Male SD rats, 18–20 months old (ii) Control: no treatment/*n* = 20 (iii) HIR only/*n* = 20 (iv) HIR+EA/*n* = 20	EA, 30 min/day, 7 days before surgery	DU20, ST-36, PC6, LI11	(i) EA improved performance in MWM (ii) EA reduced the levels of A*β* and p-tau (181) in cerebrospinal fluid (iii) EA increased the levels of *β*-catenin, Wnt, and GSK-3*β* in the hippocampus

Feng et al./2017 [121]	Hepatolobectomy (HT)	(i) Male SD rats, 1 month old (ii) Young control: no treatment/*n* = 10 (iii) D-Galactose-induced aged (DA) group/*n* = 10 (iv) DA+HT/*n* = 30 (v) DA+HT+EA for 1/3/7 days (3 groups, *n* = 10 in each group)	EA, 30 min/day after surgery, for 1, 3, or 7 days	DU20, DU14	(i) EA improved performance in Y-maze (ii) EA reduced levels of Ang II and AT1R in the hippocampus

Feng et al./2017 [36]	Partial hepatectomy (PH)	(i) Male SD rats, 21–23 months old (ii) Control: saline i.p. injection/*n* = 20 (iii) PH only/*n* = 20 (iv) PH+EA/*n* = 20 (v) PH+minocycline/*n* = 20	EA, 30 min/day after surgery, once every 2 days for 3 or 7 days	DU20, GV14	(i) EA improved performance in MWM (ii) EA reduced the levels of the proinflammatory cytokines IL-1*β*, IL-6, TNF-*α*, and HGMB1 in the hippocampus (iii) EA decreased the expression levels of TLR 4/2 in the hippocampus

Liu et al./2017 [37]	Partial hepatectomy (PH)	(i) Male SD rats, 18–20 months old (ii) Sham/*n* = 30 (iii) PH only/*n* = 30 (iv) PH+EA/*n* = 30	EA, 30 min preoperative to the end of surgery+30 min/day for 7 days after surgery	DU20, PC6, LI4	(i) EA improved performance in MWM (ii) EA increased hippocampal expression of *α*7-nAChR and downregulated TNF*α* and IL-1*β*

Abbreviations: PH: partial hepatectomy; AMIR: acute myocardial ischemia reperfusion; ST: splenectomy; TN: trigeminal neuralgia; HIR: hepatic ischemia reperfusion; HT: hepatic lobectomy; SD: Sprague Dawley; i.p.: intraperitoneal; EA: electroacupuncture; MWM: Morris water maze; IL-1*β*: interleukin-1*β*; IL-6: interleukin-6; TNF-*α*: tumor necrosis factor-*α*; MDA: malondialdehyde; SOD: superoxide dismutase; Iba-1: ionized calcium-binding adaptor molecule-1; *α*7-nAChR: *α*7-nicotinic acetylcholine receptors; p-GSK-3*β*: phosphorylated glycogen synthase kinase-3*β*; A*β*-24: amyloid *β*-protein-42; p-AMPK: phosphorylated adenosine 5′ monophosphate-activated protein kinase; NF-*κ*B: nuclear factor *κ*B; Ang II: angiotensin II; AT1R: angiotensin II type 1 receptor; Bcl-2: B-cell lymphoma/leukmia-2; Bax: Bcl-associated x protein; fEPSP: field excitatory postsynaptic potential; CHR: corticotropin-releasing hormone; ACTH: adrenocorticotropic hormone; LTP: long-term potentiation; HGMB1: high mobility group protein B1; TLR: toll-like receptor.
